# Supplementary material for: The Impact on Survival and Morbidity of Portal–Mesenteric Resection During Pancreaticoduodenectomy for Pancreatic Head Adenocarcinoma: A Systematic Review and Meta-Analysis of Comparative Studies
Source: Cancers (Basel). 2020 Jul 20;12(7):1976. doi: 10.3390/cancers12071976 (PMC7409306; doi:10.3390/cancers12071976)
Supplement: Supplementary file 1 [file cancers-12-01976-s001.pdf]

Supplementary Materials: The Impact on Survival and Morbidity of Portal–Mesenteric Resection During Pancreaticoduodenectomy for Pancreatic Head Adenocarcinoma: A Systematic Review and Meta-Analysis of Comparative Studies

Alessandro Fancellu, Niccolò Petrucciani, Alberto Porcu, Giulia Deiana, Valeria Sanna, Chiara Ninniri, Teresa Perra, Valentina Celoria and Giuseppe Nigri

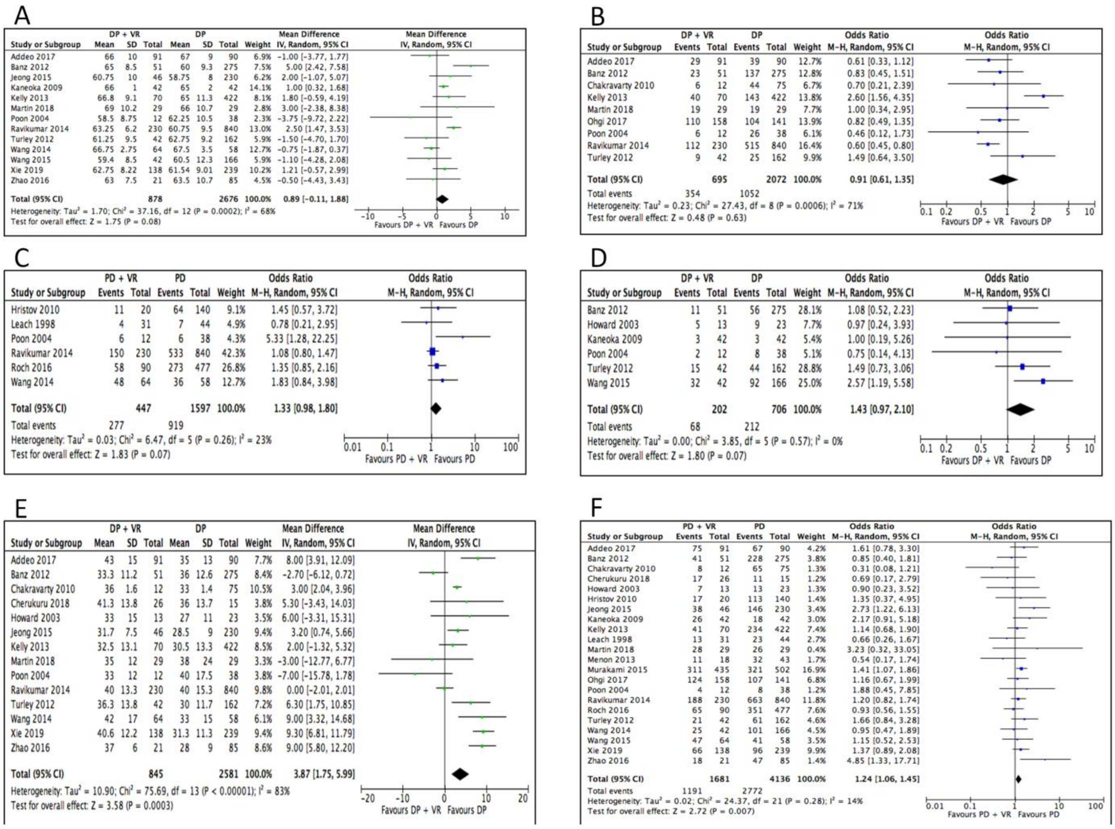

Figure S1. Meta-analysis of patients and tumor characteristics. (**A**) Age. (**B**) Need for preoperative biliary drainage. (**C**) Lymphovascular invasion. (**D**) Poorly differentiated. (**E**) Tumor size. (**F**) Positive lymph nodes.

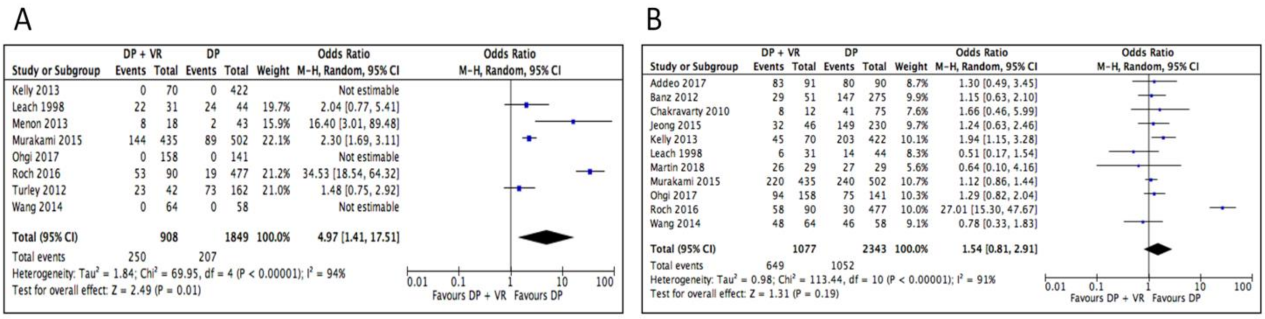

Figure S2. Meta-analysis of use of neoadjuvant chemotherapy (**A**) and adjuvant chemotherapy (**B**).

Table S1. Full electronic search strategy used for online databases and data extraction, inclusion and exclusion criteria.

Keyword used and combined for the search*	PubMed**	Scopus**	WOS**
Pancreaticoduodenectomy	8.555	16.402	5.956
Duodenopancreatectomy	8.674	743	362
Whipple OR Kaush-Wipple operation	3.864	1.131	4.368
Venous resection	9.506	9.650	6.124
Portal resection	5.209	10.905	7.007
Mesenteric vein resection	1.084	2.365	1.054
Portal-mesenteric OR porto-mesenteric resection	92	32	106
Pancreatic carcinoma	4.990	38.942	30.095
Pancreatic head carcinoma	2.710	3.680	2.910

* systematic literature search using the specified databases was performed in December 2019. **number of hits

Inclusion criteria:

- Studies comparing surgical outcomes and survival outcomes of PD with portal-mesenteric vein resection (PD+VR) and standard PD in patients with PHAC.
- Studies containing a previously unreported patient group.

Exclusion criteria:

- Studies including tumors other than pancreatic adenocarcinoma (such as ampullary carcinoma, cholangiocarcinoma, primitive duodenal cancer, and malignant neuroendocrine tumors);
- Studies including pancreatic cancer treated with total pancreatectomy or distal pancreatectomy;
- Studies including patients in whom also arterial resection was carried out along with portal-superior mesenteric resection.

Table S2. Critical appraisal of included studies using Newcastle Ottawa scale.

Study, year	Stars number			
	Selection[a]	Comparability[b]	Outcome[c]	Total
Leach, 1998 [31]	4	0	3	7
Howard, 2003 [29]	4	0	3	7
Poon, 2004 [36]	3	0	3	6
Kaneoka, 2009 [26]	4	0	4	8
Toomey, 2009 [39]	2	0	3	5
Chakravarty, 2010 [24]	4	0	3	7
Hristov, 2010 [28]	3	0	3	6
Banz, 2011 [23]	3	0	3	6
Turley, 2012 [40]	3	0	3	6
Kelly, 2013 [27]	3	0	3	6
Menon, 2013 [33]	3	0	3	6
Wang, 2014 [41]	4	0	3	7
Ravikumar, 2014 [37]	4	0	3	7
Wang, 2015 [8]	3	0	3	6
Jeong, 2015 [30]	3	0	3	6
Murakami, 2015 [34]	4	0	3	7
Zhao, 2016 [43]	3	0	3	6
Roch, 2016 [38]	3	0	3	6
Ohgi, 2017 [35]	3	0	3	6
Addeo, 2017 [12]	4	0	3	7
Martin, 2018 [32]	4	1	3	8
Cherukuru, 2018 [25]	3	0	3	6
Xie, 2019 [42]	3	1	3	7

[a] Maximum 4 stars; [b] Maximum 2 stars; [c] Maximum 3 stars.

Table S3. Type of venous resection and reconstruction performed in the patients of the PD+VR group.

Author	Type of Venous resection and Venous Reconstruction
Addeo P [12]	End-to-end anastomosis: 91 (100%)
Banz VM [23]	Primary anastomosis: 48 (94.1%)<br>Donor vein: 2 (3.9%)<br>Autologous vein: 1 (2%)
Chakravarty K [24]	End-to-end anastomosis: 9 (75%)<br>Autologous graft: 1 (8.3%)<br>PTFE graft: 2 (16.7%)
Cherukuru R [25]	Segmental resection: 25 (96.2%)<br>Sleeve resection: 1 (3.8%)<br>Primary anastomosis: 12 (46.2%)<br>Autologous vein graft: 10 (38.5%)<br>PTFE graft: 3 (11.5%)
Howard TJ [29]	End-to-end anastomosis: 6 (46%)<br>Lateral venorrhaphy: 5 (38%) [one with saphenous vein patch]<br>Resection and interposition vein graft 2 (15%)
Hristov B [28]	Primary closure: 12 (60%)<br>End-to-end anastomosis: 6 (30%)<br>Interposition grafts: 2 (10%) [one autologous saphenous vein; one PTFE graft]
Jeong J [30]	Partial vein resection with primary closure: 12 (26.1%)<br>Segmental resection with anastomosis: 34 (73.9%) [1 cadaveric vein graft; 1 autologous saphenous vein graft]
Kaneoka Y [26]	End-to-end anastomosis: 27 (64.3%)<br>External iliac vein interposition graft: 15 (35.7%)
Kelly KJ [27]	Tangential or segmental vein resection: 70 (100%)

Table S3. *Cont.*

Leach SD [31]	Saphenous vein patch: 4 (12.9%)<br>End-to-end anastomosis with ligation and division of the splenic vein: 8 (25.8%)<br>Primary end-to-end anastomosis without ligation and division of the splenic vein: 7 (22.6%)<br>Internal jugular vein interposition graft: 11 (35.5%)<br>Gore-Tex interposition graft: 1 (3.2%)
Martin D [32]	End-to-end anastomosis: 20 (69%)<br>PTFE graft: 6 (21%)<br>Wedge resection with direct closure: 3 (10%)
Menon VG [33]	End-to-end anastomosis: 12 (67%)<br>Internal jugular vein graft: 1 (5.6%)<br>Renal vein graft: 2 (11.1%)<br>Synthetic graft: 3 (16.7%)
Murakami Y [34]	Wedge resection: 76 (17.5%)<br>Segmental resection: 353 (81.1%)<br>End-to-end reconstruction: 323 (91.5%)<br>Autologous vein interposition graft: 30 (8.5%)
Ohgi K [35]	Tangential wedge resection: 17 (12%)<br>Segmental resection: 125 (88%)
Poon RT [36]	Wedge resection: 4 (33.3%)<br>End-to-end anastomosis: 8 (66.7%)
Ravikumar R [37]	Primary closure: 129 (56.1%)<br>End-to-end anastomosis: 65 (28.3%)<br>Interposition graft: 36 (15.7%)
Roch AM [38]	End-to-end anastomosis: 67 (74.4%)<br>Internal jugular vein graft: 19 (21.1%)<br>Renal vein graft: 1 (1.1%)<br>Saphenous vein graft: 1 (1.1%)<br>PTFE graft: 2 (2.2%)
Toomey P [39]	Lateral venorrhaphy: 44 (91.6%)<br>Saphenous vein graft: 2 (4.2%)<br>End-to-end anastomosis: 2 (4.2%)
Turley RS [40]	Primary repair: 8 (19%)<br>Vein patch: 25 (59.5%)<br>Interposition graft: 9 (21.4%)
Wang F [41]	End-to-end anastomosis: 54 (84.4%)<br>Lateral venorrhaphy: 9 (14.1%)<br>Synthetic graft: 1 (1.6%)
Wang WL [8]	End-to-end anastomosis: 28 (66.7%).<br>Interposition venous allograft (portal veins or iliac arteries from liver transplant donors): 14 (33.3%)
Xie ZB [42]	Primary closure of the vein, and reconstructed with PTFE vascular grafts: 138 (100%)
Zhao X [43]	Interposition of Iliac vein from cadaver donor: 21 (100%)

© 2020 by the authors. Licensee MDPI, Basel, Switzerland. This article is an open access article distributed under the terms and conditions of the Creative Commons Attribution (CC BY) license (http://creativecommons.org/licenses/by/4.0/).
